# The chronobiology of human heart failure: clinical implications and therapeutic opportunities

**DOI:** 10.1007/s10741-024-10447-1

**Published:** 2024-10-11

**Authors:** Francesco Gentile, Michele Emdin, Claudio Passino, Sabrina Montuoro, Paola Tognini, John S. Floras, John O’Neill, Alberto Giannoni

**Affiliations:** 1 Health Science Interdisciplinary Center, Scuola Superiore Sant’Anna, Pisa, Italy; 2 Division of Cardiology and Cardiovascular Medicine, Fondazione Monasterio, Pisa, Italy; 3 University Health Network and Sinai Health Division of Cardiology, Toronto, ON Canada; 4 Medical Research Council Laboratory of Molecular Biology, Cambridge, UK

**Keywords:** Heart failure, Circadian rhythms, Chronobiology, Chronotherapy, Sleep medicine, Pathophysiology

## Abstract

Circadian variation in cardiovascular and metabolic dynamics arises from interactions between intrinsic rhythms and extrinsic cues. By anticipating and accommodating adaptation to awakening and activity, their synthesis maintains homeostasis and maximizes efficiency, flexibility, and resilience. The dyssynchrony of cardiovascular load and energetic capacity arising from attenuation or loss of such rhythms is strongly associated with incident heart failure (HF). Once established, molecular, neurohormonal, and metabolic rhythms are frequently misaligned with each other and with extrinsic cycles, contributing to HF progression and adverse outcomes. Realignment of biological rhythms via lifestyle interventions, chronotherapy, and time-tailored autonomic modulation represents an appealing potential strategy for improving HF-related morbidity and mortality.

Our lives revolve around time-dependent rhythms, aimed to maximize efficiency and dampen perturbations in homeostatic equilibrium induced by environmental changes [1]. The term “circadian” (from the Latin words “*circa-*” “*-diēm*,” i.e., around a day) denotes rhythms with a period approximating 24 h, generated by a biological clock and persisting in the absence of external cues [2]. The terms “ultradian” and “infradian” refer to shorter or longer cycles, respectively [2]. Different conditions affect these rhythms, including exposure to darkness and light, and the timing of sleep, meals, and exercise [3]. While homeostasis usually re-equilibrates after acute challenges (e.g., shift work or traveling across time zones), their recurrence and persistence may sustain maladaptive mechanisms in the chronic setting [3].

In recent decades, a large body of literature has highlighted the importance of cardiovascular chronobiology. In particular, (1) peripheral clocks, modulating protein synthesis, activity, and turnover in a circadian fashion are present in most cardiovascular cells [4]; (2) the neurohormonal [4], endothelial [5], and humoral signals [6] modulating cardiovascular functions show circadian rhythms; (3) dampened and/or misaligned variations in cardiovascular and respiratory functions elicit autonomic imbalance and contribute to incident cardiovascular events and/or progression of cardiovascular disorders [7]; (4) the occurrence of acute cardiovascular events shows time-dependent patterns [8]; and (5) chronotherapy, i.e., time-tailored therapeutic approaches, may improve cardiovascular outcomes [9].

Regardless of its etiology (e.g., genetic, ischemic, infective, and metabolic), chronic heart failure (HF) is characterized by the inability of the heart to augment perfusion to meet the energy requirement of tissues, or to do so at the price of increasing ventricular end-diastolic volume or filling pressure [10]. Concurrent activation of intrinsically rhythmic neuro-hormonal systems will promote adverse cardiac remodeling and augment arrhythmic potential [10]. In otherwise healthy adults, long-term disruptions in sleep onset and duration, the timing of meals and exercise, and the difference between day-time and night-time blood pressure increase the risk of developing HF [11]. Once HF is established, autonomic and hormonal rhythms often are dampened or misaligned, degrading quality of life and worsening prognosis [11].

In a comprehensive recent review of primarily pre-clinical data by Jamal et al. [12], circadian rhythms emerged as pivotal in shaping cardiac physiology across various dimensions, encompassing contractility, metabolism, and electrophysiology. Alterations in the daily variations of protein abundance, activity, and turnover, documented in preclinical studies, have been identified as a precursor to HF development [12]. The relevance of such findings to HF in humans is as yet only partially explored. The impetus for this brief review is, therefore, the recognition that a more profound comprehension of mechanisms responsible for and consequences of disturbed chronobiology in HF patients may yield clinical insights relevant to management, through the exploration of novel therapeutic targets. In this respect, this review will introduce the potential role of time-specific lifestyle modifications and medication administration, innovative drugs targeting specific chrono-related targets, and refinement of bio-electronic medicine algorithms.

Considering the lack of robust clinical studies confirming the clinical relevance of these mechanisms in HF management, the scope of this hypothesis-generating review is to provide a comprehensive overview of the current evidence regarding the potential role of chronobiology in HF pathophysiology, encompassing aspects such as the risk of incident HF, disease progression, and patient outcomes.

## Molecular mechanisms

Molecular mechanisms underpinning the circadian regulation of human cardiovascular rhythms are only partially understood.

The suprachiasmatic nucleus (SCN) of the hypothalamus is considered the main regulatory center of circadian rhythms, synchronizing cells within tissues/organs to adapt body functions to temporal shifts [13]. To do so, light/dark signals, sensed by retinal cells, converge to the SCN, where they are integrated with cortical/subcortical and peripheral afferent inputs [13]. The final output is conveyed into downstream neural pathways and neurohormonal cascades, modulating cardiovascular function through the autonomic nervous systems and neurohormones such as cortisol and melatonin [13, 14].

Appreciation that cardiovascular rhythms are evident in isolated cardiovascular cells has prompted investigation concerning the presence and roles of peripheral clocks responsible for the modulation of cellular processes to anticipate internal changes and/or external stimuli [15]. Feedback-regulated transcriptional, translational, and post-translational loops have been identified in cardiovascular cells, as in all cell types in mammals except stem cells. The core clock genes driving circadian expression include the circadian locomotor output cycle kaput (CLOCK), brain and muscle Arnt-like protein-1 (BMAL1), cryptochrome (CRY), and period (PER) genes, with the products of the orphan nuclear-receptor gene (Rev-erbα) and retinoic acid receptor-related orphan receptors α/β (RORα/β) acting as modulators [16]. The rhythmic expression of these genes is crucial in cardiovascular cells, regulating up to 10% of the genome [17]. Of note, post-transcriptional and post-translational mechanisms, such as protein sequestration, translocation, and turnover, primarily mediated by the mammalian target of rapamycin complex (mTORC), seem to play some role as well in the circadian regulation of cellular functions [18]. However, the specific roles of these additional mechanisms in cardiovascular cells remain to be fully elucidated.

Circadian rhythms affect different functions in cardiac cells, including contractility, metabolism, ion channel expression, and response to injury [17]. This is not surprising considering the overall complexity of the heart, in which the cellular organization within a functional syncytium is fundamental for synchronized contraction and efficient pumping [11].

To do so, both the central and peripheral clocks should adapt body functions to environmental changes. The term “*zeitgeber*” (i.e., time-giver or synchronizer) refers to the endogenous and exogenous cues that could influence, or entrain, circadian rhythms [19]. While light/dark signals synchronize the activity of the SCN, food intake, physical exercise, and body temperature are the main *zeitgebers* for peripheral clocks [11]. In this respect, by synchronizing multi-hormonal signals and body temperature, feeding time affects substrate utilization, metabolic flexibility, and energetic efficiency [20]. Similarly, physical exercise entrains hormonal fluctuation, autonomic activity, substrate availability, and body temperature [21]. Finally, also shifts in body temperature affect biological rhythms in mammalian cells, though the functional consequences remain unclear [22].

## Abnormal biological rhythms and risk of incident heart failure in the general population

**Fig. 1:**
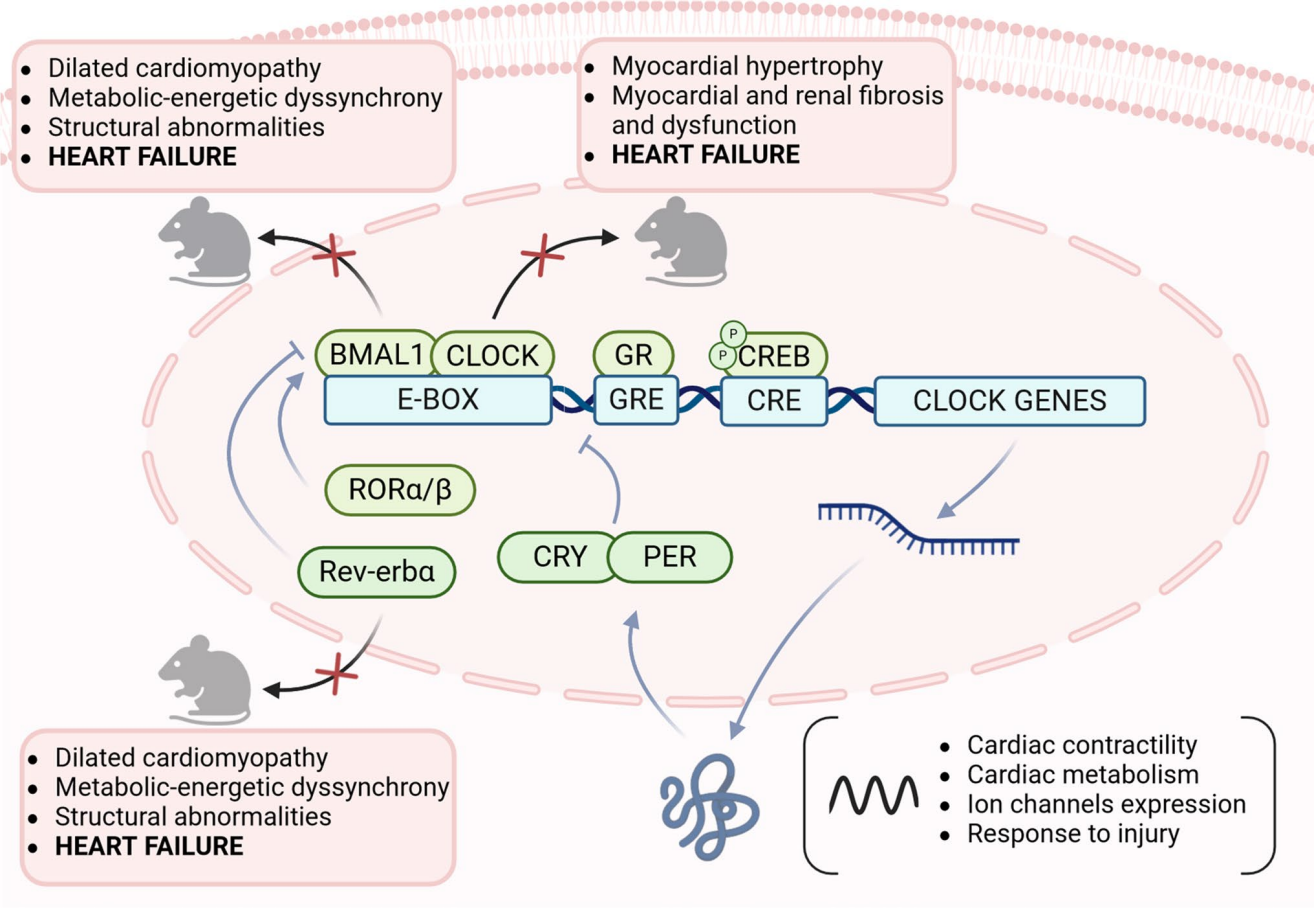
Clock genes disruption and heart failure development: evidence from animal studies. By using knockout models, several preclinical studies have shown how the disruption of the molecular clock within the cardiomyocyte may promote heart failure development [25–31]. Inefficient metabolism, structural abnormalities, and adverse cardiac remodeling have been identified as the involved mechanisms. BMAL1: brain, and muscle Arnt-like protein 1; cAMP: cyclic adenosine-monophosphate; CLOCK: circadian locomotor output cycle kaput; CRE: cAMP response element; CREB: cAMP response element-binding protein; CRY: cryptochrome; GR: glucocorticoid receptor; GRE: glucocorticoid regulatory element; PER: period; Rev-erbα: orphan nuclear-receptor gene; RORα/β: retinoic acid receptor-related orphan receptors α/β

The importance of cardiac peripheral clocks has been outlined in preclinical studies, showing a close association between the knockout of clock genes and HF development, as extensively reviewed elsewhere [12, 23, 24] and summarized in Fig. 1 [25–30]. Furthermore, pivotal animal models showed that the misalignment between endogenous and exogenous rhythms predisposes to HF development as well, mainly involving the SCN [32, 33]. Indeed, through a multisynaptic connection [34], the SCN entrains cardiovascular rhythms by modulating the cross-talk between the two branches of the autonomic nervous system, as reflected in heart rate variability, which shows circadian oscillations in healthy individuals, with a predominance of parasympathetic activity at night, and an early-morning peaking of sympathetic activity [4]. The loss of synchrony between these physiological oscillations and other endogenous (e.g., hormonal cascades, metabolic functions) and/or exogenous (e.g., the timing of sleeping, feeding, and exercising) rhythms may hence promote maladaptive mechanisms leading to HF development.

Most evidence connecting abnormal daily rhythms to HF development in the general population derives from observational investigations. Clinical studies evaluating candidate mechanisms, including neurohormonal imbalance [7], endothelial dysfunction [35], pro-inflammatory, and pro-fibrotic cascades [35], have related alteration of their rhythmicity to an increased risk of incident HF.

In healthy individuals, sleep deprivation and insomnia have been consistently related to a higher long-term risk of HF [36–38]. In a large cohort (*n* = 12,761) of middle-aged and older adults free from HF at baseline, the presence and cumulation of insomnia symptoms (i.e., difficulty initiating sleep, difficulty maintaining sleep, early-morning awakening, and non-restorative sleep) were associated with an up to 80% higher risk of incident HF [36]. In a large genome-wide association study, involving more than 2 million participants without history of HF, self-reported daytime napping, insomnia, and short sleep duration were causally associated with a high risk of HF [39]. Furthermore, delayed bedtimes and wake-up times on weekdays were associated with an increased risk of incident HF in a multicenter prospective cohort study involving 4765 participants with an 11-year follow-up [37]. Of note, in another study, of 12,268 twins, the impact of abnormal sleeping patterns (i.e., napping, sleeping < 7 or ≥ 10 h over the 24 h) on cardiovascular risk was not affected by genetic or environmental confounders [38].

By altering sleep/wake cycles, obstructive sleep apnea (OSA) represents an important source of circadian disruption in the general population, promoting incident HF [40]. In a seminal study by Gottlieb et al. (*n* = 4422), men with severe OSA were 58% more likely to develop HF than those without OSA over a median 8.7-year follow-up [41]. Secondary to the periodic collapse of upper airways due to anatomical and/or functional mechanisms during sleep, OSA is associated with abnormal sleep architecture and daytime sleepiness [40]. Furthermore, desaturation-related chemoreflex stimulation and arousals promote nighttime adrenergic surges and neurohormonal activation [40]. Through these mechanisms, OSA contributes to altering blood pressure rhythms (which physiologically decrease during the night), underlying the hypertensive phenotypes characterized by nocturnal hypertension (namely, non-dipper and reverse-dipper) [42]. Importantly, nocturnal hypertension is strongly associated with HF risk. Among 951 individuals without HF at baseline, a non-dipper profile was associated with a higher HF risk over a median 9-year follow-up [43]. These findings were confirmed in 6359 individuals in which non-dipper and, particularly, riser phenotypes were associated with over twofold higher HF risk during a mean 4-year follow-up, independently of 24-h, daytime, and nighttime blood pressure [44].

Beyond sleep disorders, also exposure to abnormal exogenous rhythms may predispose to HF development. In a study involving 242,754 individuals from the UK Biobank, permanent shift work was associated with a higher risk of incident HF; this association was significant only in females [45]. Similarly, exposure to unphysiological light/dark cycles, in particular during winter, was also associated with higher HF-related mortality in people living at extreme latitudes [46]. The impact of other potential rhythm disruptions (e.g., recurrent jetlag, artificial light exposure, and noise pollution) on the risk of incident HF is plausible but remains to be investigated.

The incidence of HF after acute coronary syndromes also shows circadian variability. Among 3625 patients with acute myocardial infarction, new-onset HF was more frequent in those admitted at night (17% vs. 10% at daytime) [8]. These observation were corroborated by findings in a cohort of 6710 patients, in whom infarct area extension and acute HF incidence were greater in those admitted between midnight and 6 a.m. [47]. Supported by pre-clinical evidence [48], the authors proposed a role for the time-dependent oscillations in repair mechanisms and ischemia/reperfusion damage as a possible explanation [47]. Leucocyte recruitment to arteries, mediated by sympathetic innervation, shows circadian rhythmicity[49]. The influence of the time of the day on the efficiency of healthcare organization systems and operators is likely also contributory [50].

## Abnormal biological rhythms, disease progression, and outcomes in patients with overt heart failure

The knockout of clock genes has been associated with HF development in animal models [12]. On the other hand, the role of the peripheral clocks in HF progression is uncertain since (1) the expression of clock genes is generally preserved in established HF and (2) the circadian oscillations in protein abundance, sequestration, and turnover in cardiac cells remain to be assessed in HF models [18].

**Fig. 2:**
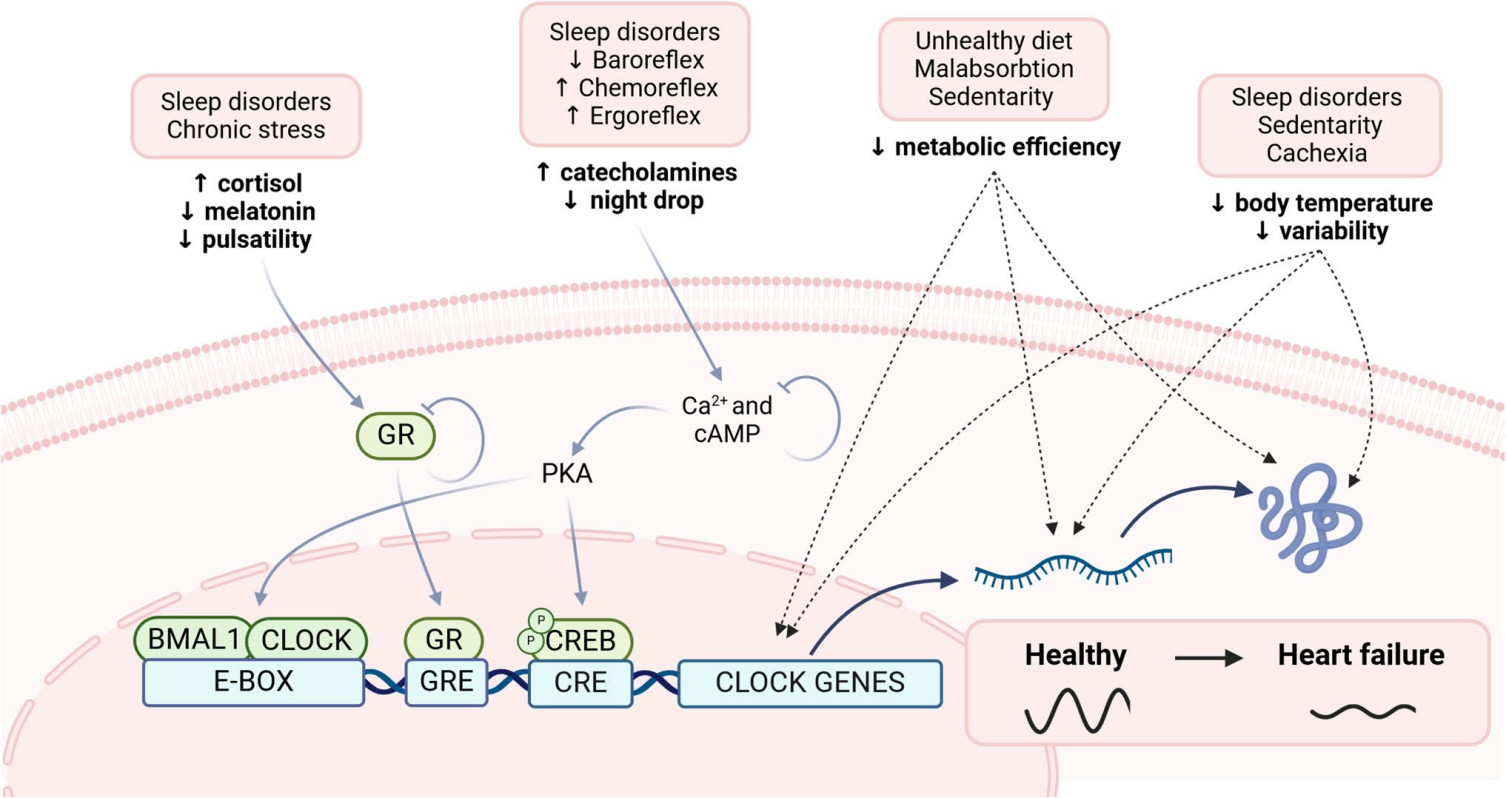
Abnormal biological rhythms in heart failure patients. Biological rhythms are often altered in heart failure patients through different mechanisms. Though sleep disorders and autonomic imbalance, with a predominance of sympathetic over parasympathetic activity, have been largely demonstrated, the related effects on the peripheral clock have been less explored. Similarly, dietary habits, malabsorption, and a sedentary lifestyle contribute to altering biological rhythms, by modulating peripheral clocks. BMAL1: brain, and muscle Arnt-like protein 1; cAMP: cyclic adenosine-monophosphate; CLOCK: circadian locomotor output cycle kaput; CRE: cAMP response element; CREB: cAMP response element-binding protein; GR: glucocorticoid receptor; GRE: glucocorticoid regulatory element

Dampened and/or misaligned cardiovascular rhythms, secondary to neurohormonal imbalance, have been observed in patients with chronic HF, contributing to adverse remodeling, disease progression, and poor outcomes [51]. Altered sleep/wake cycles, breathing disorders, and sedentary lifestyles are some possible causes (Fig. 2). Interestingly, neurohormonal signals also exert a direct influence on peripheral clocks. Indeed, the acute exposure of rats’ cardiomyocytes to norepinephrine, mimicking a burst of sympathetic activity, induced oscillations in clock gene expression (BMAL1, REV-ERBα, and PER2) [23]. However, how the chronically elevated sympathetic activity characterizing HF patients affects peripheral clock functions remains to be investigated. In general, the study of the impact of altered circadian rhythms and endogenous and exogenous cues, due to altered sleep, food malabsorption for bowel congestion, reduced thermal variation due to decreased mobility and HF progression is a topic of great interest that should be systematically studied in the future.

### Cardiac peripheral clocks in heart failure

In animal HF models, the circadian expression of most clock genes is preserved, with only minor differences in single clock gene expression levels or acrophase [6]. Similarly, a circadian expression of PER1, PER2, and BMAL1 was preserved in candidates for cardiac transplantation, while no oscillation was detected for CRY1, with no significant difference among patients with ischemic (*n* = 16) or nonischemic (*n* = 30) cardiomyopathies, or heart-healthy controls (*n* = 9) [52]. In a more recent study, a circadian pattern of expression was observed in patients undergoing cardiac transplantation, but not in brain-dead donors, for both clock genes (ARNTL, DBP, NFIL3, NR1D1, and PER2) and ion channel genes (e.g., SCN5A, KCNA5, KCNB1, SLC8A1, and KCNA4) [53], underscoring a potential selection bias for “controls” in human studies.

The sole evidence of abnormality in peripheral clocks in humans arises from a retrospective analysis of explanted hearts from patients with dilated cardiomyopathy undergoing transplantation (*n* = 36) [29]. In this study, Song et al. observed a relation between REV-ERBα/BMAL1 expression ratio and cardiac remodeling [29]. Two different chronotypes were identified in HF patients according to the relative expression of REV-ERBα and BMAL1 compared to normal hearts (*n* = 6). Patients with a higher REV-ERBα/BMAL1 ratio in the morning and a lower ratio in the afternoon (“chronotype B,” *n* = 16) had more dilated left ventricles and severe mitral regurgitation compared with those with a lower ratio in the morning and a higher ratio in the afternoon (“chronotype A,” *n* = 20), independent of age, sex, body mass index, and medications [29].

Despite being explorative, these findings support a possible role for the peripheral clocks within the cardiomyocyte in the pathophysiology of HF.

### Abnormal neurohormonal rhythms in heart failure

Autonomic imbalance is a key pathophysiological determinant in patients with over HF, in which it is associated with adverse remodeling, arrhythmogenesis, and poor outcomes [54]. The importance of the abnormal circadian modulation of autonomic cardiovascular control in HF patients emerged from landmark physiology studies. Reduced heart rate variability was indeed observed from the mild stages of the disease and associated with mortality [51].

Alterations in the circadian rhythms of cardiovascular autonomic reflex arcs (e.g., baroreflex and chemoreflex) are possible contributors [54, 55]. While baroreflex sensitivity is physiologically higher at night in healthy subjects, when parasympathetic activity prevails and adrenergic surges should be blunted, this circadian nighttime increase of baroreflex function seems lost in HF patients, particularly in the 50% or more who also have sleep-disordered breathing [56]. Similarly, while chemoreflex response is lower at night in healthy individuals, to blunt ventilatory and adrenergic surges [57], the circadian modulation of chemoreflex sensitivity was reduced in a rat model of HF, promoting breathing instability and autonomic imbalance [58]. Considering their pathophysiological and prognostic role in patients with HF [59], baroreflex [60] and chemoreflex sensitivity [61] are emerging as therapeutic targets. A deeper study of their circadian aspects may help maximize the benefit of tailored therapies and reduce undesired effects.

The molecular pathways linking abnormal autonomic rhythms to HF progression are less understood and mainly derived from preclinical evidence. In a post-myocardial infarction mouse model of HF, the blunted oscillations in baroreflex sensitivity were related to an upregulation of the brainstem expression of angiotensin-II type-1 receptor and oxidant pathways [62]. In a similar model, an intertwined neuronal pathway connecting central autonomic centers to the SCN and the heart, through the superior cervical ganglion and paraventricular nucleus of the hypothalamus, was identified by trans-synaptic tracing [63]. In this respect, sympathetic denervation of the pineal gland, mediated by the fibrosis and macrophagic infiltration of the superior cervical ganglion, was observed in a mouse HF model (transverse aortic constriction) and confirmed by analyzing post-mortem specimens of 10 HF patients [64].

Beyond autonomic nervous systems, the abnormal rhythms of other hormonal systems contribute to HF pathophysiology [65]. Glucocorticoids modulate peripheral clocks, affecting the expression of clock genes by binding to a specific DNA promoter region [66]. In HF patients, the physiological pulsatile release of cortisol is dampened [6, 67], and increased cortisol levels held incremental prognostic value [68]. A chronic nonspecific stress response may be a possible explanation [67].

Abnormal sleep/wake cycles and breathing disorders have been proposed as the main determinants of the alteration of neurohormonal rhythms in patients with HF.

Over half of HF patients exhibit abnormal breathing patterns, encompassing OSA and central apneas (CA), which strongly contribute to the disruption of sleep/wake cycles [69]. Apnea and hypoxia severity and sleep fragmentation may be augmented by recumbent sleep posture, resulting in rostral fluid shift into the thorax and jugular veins [70, 71]. Fluid redistribution, together with salt retention and renal dysfunction, contributes to the abnormal day/night blood pressure rhythms in HF patients, via both hemodynamic and neurohormonal mechanisms [72]. The lack of the nocturnal blood pressure dip is more frequent in HF patients compared to healthy controls, proportionally to disease severity [73, 74], with up to 77% of patients, in one study, classified as non-dippers [6]. Importantly, in a cohort of 110 patients with HF and reduced ejection fraction, both non-dipper and reverse-dipper status were associated with a higher risk of death or HF-related hospitalization [75]. Although CA may be observed also during wakefulness in a subset of HF patients, they are usually more severe during sleep [76, 77]. Of note, compared to matched controls and HF patients with stable breathing, HF patients with CA had significantly shorter sleep latency and a higher frequency of arousals [78]. The disruption of sleep stages with longer stages 1 and 2 non-rapid eye movement sleep and less rapid eye movement sleep was identified as an involved mechanism [78].

As extensively reviewed elsewhere [76, 77, 79], both OSA and CA are associated with intermittent desaturations and chemoreflex stimulation, promoting neurohormonal imbalance, oxidative and inflammatory pathways, and abnormal metabolic cascades, with adverse clinical and prognostic consequences in HF patients. A key role in this context has been attributed to hypoxemic burden, which contributes to altering sleep architecture and autonomic balance and has been identified as a robust and independent predictor of mortality [80, 81]. Nevertheless, as detailed below, improving outcomes by targeting sleep-disordered breathing represents an unmet need in cardiology.

### Cardiac metabolism, skeletal muscle, and body temperature in heart failure

Food intake, physical exercise, and body temperature represent key *zeitgebers* for cardiac peripheral clocks. Though less extensively explored, alterations in their physiological rhythms may retain pathophysiological significance in patients with chronic HF.

Abnormal metabolic processes characterize the failing heart [82]. Similarly to other tissues, cardiomyocytes show daily metabolic oscillations to anticipate energetic demands [83]. Food intake is a physiological *zeitgeber* and both insulin and insulin-growth factor-1 entrain peripheral clocks [84]. While the experimental disruption of clock genes resulted in abnormal cardiac metabolism [29, 85], no studies have investigated the interaction between abnormal circadian rhythms and cardiac metabolism in HF so far. Similarly, whether specific nutritional patterns may modulate, positively or negatively, metabolic rhythms in HF patients is currently unknown. Interestingly, gut physiology and microbiome seem to be involved in these pathways and may be possible targets [86].

The expression of several genes in the skeletal muscles, encoding for both structural and enzymatic elements exhibits circadian oscillations, and physical activity is a *zeitgeber* for peripheral clocks [21]. In patients with chronic HF, physical activity is often reduced, due to clinical conditions and deconditioning [87]. The key role of skeletal muscles in HF has been outlined in the so-called “muscle hypothesis.” Accordingly, reduced peripheral perfusion, together with inflammatory and neurohormonal activation, establish a systemic myopathy, contributing to limiting symptoms and eliciting sympathetic activity through an overactive ergoreflex response [87]. A sedentary lifestyle, along with abnormal neurohormonal signals and wasted sleep/wake cycles, may hence alter the physiological modulation of skeletal muscle function, impairing energetic efficiency, and sustaining autonomic imbalance.

Since body temperature shows physiological circadian rhythms and entrains peripheral clocks [19], the alteration of its variability may hold adverse consequences in HF. In cardiomyopathic hamsters, a significant decrease in the amplitude of body temperature oscillations was observed 8 weeks before death, preceding all other evidence of decompensation [88]. Interestingly, among 198 patients hospitalized for decompensated HF, decreasing body temperature was associated with a significantly higher risk of readmission and death [89]. Although a causal relationship was not established, an interaction between neuroendocrine and immune systems was postulated [89].

## Chronobiology-stimulated interventions in heart failure

**Fig. 3:**
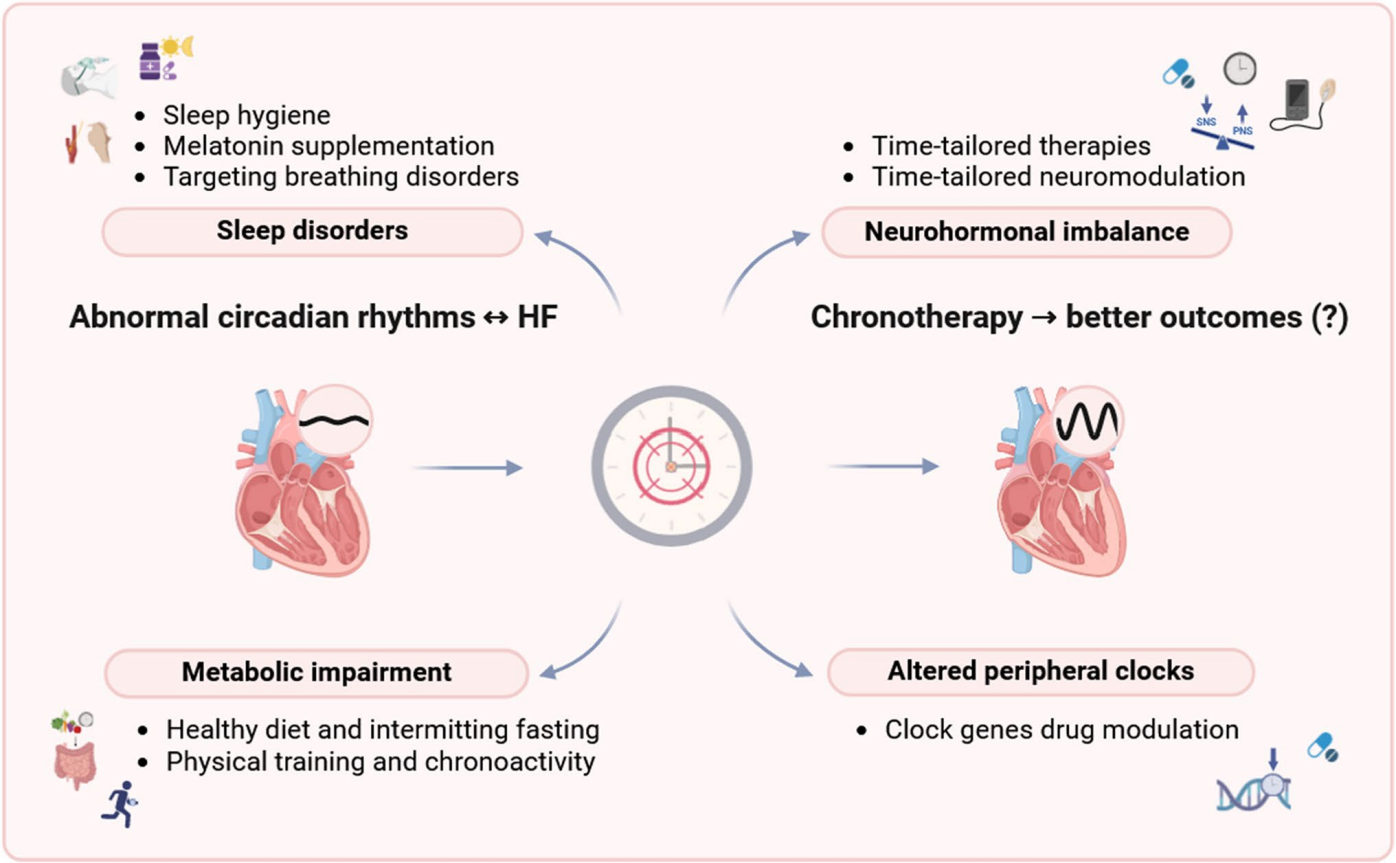
Chronobiology and heart failure: therapeutic opportunities. Biological rhythms are often altered in patients with heart failure (HF), contributing to disease severity, and clinical and prognostic aggravation. The realignment of endogenous and exogenous rhythms by acting on sleep disorders, neurohormonal imbalance, energetic inefficiency, and, potentially, peripheral clocks may improve HF-related morbidity and mortality. PNS: parasympathetic nervous system; SNS: sympathetic nervous system

Only a few studies have investigated the clinical and prognostic impact of restoring biological rhythms in HF patients. Below, some promising approaches under investigation are briefly reviewed (Fig. 3).

### Chronotherapy

The term “chronotherapy” refers to the therapeutic application of chronobiological knowledge. Though rarely integrated into current clinical practice, this approach may be relevant for cardiovascular pharmacotherapy. Many of the pathophysiological mechanisms involved in the development and progression of cardiovascular diseases identified as drug targets exhibit circadian variation (e.g., heart rate, blood pressure, activation of the sympathetic nervous system and renin–angiotensin–aldosterone axis, leukocyte migration into arteries, and platelet function) [90]. Also, several genes encoding for proteins involved in drug transport and metabolisms, as well as pharmacological targets show circadian regulation [90]. Adapting the timing of drug administration to these biological rhythms may thus prove efficacious.

Chronotherapy may prove valuable in preventing HF development by optimizing the management of systemic hypertension. In a mouse model of pressure overload-related HF, the sleep-time administration of an angiotensin-converting-enzyme inhibitor resulted in less severe cardiac remodeling and improved contractility, compared to both placebo and wake-time therapy [9]. Though not tested in humans so far, these findings are in line with those of a sub-analysis of the placebo-controlled HOPE study, in which the bedtime administration of ramipril resulted in a significant reduction of all major adverse cardiovascular events including all-cause mortality in high-risk patients, despite only a marginal change in daytime blood pressure [91]. Interestingly, in a subgroup of patients undergoing 24-h ambulatory blood pressure monitoring, a significant improvement in 24-h and nighttime blood pressure control emerged and was proposed as a possible explanation for this mortality benefit [92]. More recently, the TIME study, randomizing 24,610 hypertensive patients to take their antihypertensive medications in either the morning or in the evening, failed to show a significant difference between the two arms in preventing HF-related outcomes [93]. However, the dipping status of the enrolled patients was unknown and not a trial entry criterion; its open-label design and use of different drugs were other trial limitations [93]. Testing this approach in a cohort of non-dippers or reverse dippers may yield a different outcome [94].

Chronotherapeutic strategies remain unexplored in patients with chronic HF. Nocturnal hypertension is associated with a higher risk of adverse events in HF patients [75], but in those with a preserved nocturnal dip, further blood pressure lowering during sleep may worsen organ perfusion. While many of the drugs recommended for HF management are administered twice daily (including sacubitril/valsartan, some beta-blockers, ACE-inhibitors, and angiotensin-receptor-blockers) [95], the potential influence of time-of-the-day on efficacy and adverse effects could be explored for other agents administered once-daily (e.g., mineralocorticoid-receptor-antagonists, bisoprolol, and nebivolol). Interestingly, gliflozins, which have recently revolutionized HF therapy, positively affected the circadian control of the autonomic nervous system in rats with metabolic syndrome [96].

In healthy volunteers, the positive effects of transcutaneous vagus nerve stimulation on heart rate variability were greater if the stimulus was delivered in the morning than in the evening [97]. Whether these effects may be replicated in HF patients, characterized by reduced heart rate variability, remains to be investigated. Residual sympatho-vagal imbalance persists in many HF patients receiving guideline-directed medical therapy [98]. Devices such as baroreflex activation therapy and vagus nerve stimulation rely on open-loop algorithms that as yet do not permit dose–effect or time-of-the-day adjustment [99]. Restoration of their rhythmicity for example, by synchronizing neuromodulation to time and activity may maximize their prognostic benefits [100].

Further inhibitory modulation of sympathetic nerve activity may be a complementary or alternative strategy. In a mouse model of myocardial infarction, circadian disruption-related cardiac remodeling was associated with increased sympathetic activity and attenuated by its inhibition, obtained through pharmacological ganglionic blockade [63]. In contrast, counteracting irreversibly sympathetic surges through surgical or trans-catheter denervation overlooks daily variations [101]. Novel drug delivery systems or closed-loop bio-electronic devices designed for intermittent modulation of sympathetic activity at the central or ganglionic level are intriguing strategies for future investigation [99].

### Improving sleep quality and treating breathing disorders

Reduced serum levels of melatonin have been associated with abnormal sleep/wake cycles and increased HF incidence and progression [102]. Therefore, its supplementation may provide positive effects either by improving sleep/wake rhythms or through its antioxidant, anti-apoptotic, and anti-fibrotic properties [103]. Following encouraging preclinical evidence [103], in a double-blind phase 2 randomized controlled trial, 85 HF patients with reduced ejection fraction were randomized to a 24-week treatment with 10 mg melatonin vs. placebo [104]. Melatonin supplementation was associated with lower N-terminal pro-B-type natriuretic peptide concentration, improved quality of life, and reduced symptoms at follow-up [104]. No differences were observed in terms of sleep quality and echocardiographic parameters [104]. Considering the small sample size and the monocentric design, further studies in larger populations are required to extend these findings [104].

Reducing the burden of breathing disorders may restore sleep/wake rhythms, by improving sleep architecture and autonomic equilibrium. Accordingly, in the general population, treating OSA improved sleep quality and autonomic balance [105]. Nevertheless, conflicting findings have been obtained when analyzing hard outcomes, including incident HF [105]. While reducing the nighttime burden of OSA and CA improved sleep quality, quality of life, autonomic balance, and remodeling also in HF patients, several controlled studies testing nighttime mask-based systems conducted failed to demonstrate significant benefit on HF-related hospitalization or mortality [106, 107].

Different from OSA, CA are not only sleep-related, and their daytime occurrence has been associated with worse prognosis [76, 77]. Targeting the pathophysiological determinants of CA over 24 h may be a more rational alternative. In this regard, chemoreceptors are emerging as intriguing targets [61]. As for peripheral chemoreflex, the antagonism of the purinergic P2X3-receptor, overexpressed in the carotid bodies, decreased chemoreflex-mediated sympathetic activity in hypertensive rats [108] and the incidence of apneas of prematurity in newborn rats [109], and could now be tested in humans. On the other hand, the administration of the 5HT_1A_ receptor agonist buspirone, acting on the central chemoreceptors, was effective in reducing CA and chemoreflex sensitivity in chronic HF patients, with no adverse effects [110]. While future studies should confirm these exciting preliminary findings, testing the impact of these molecules on the chronobiology of chemoreflex function (altered in preclinical models of HF) [58] may help to refine and optimize their clinical application.

### Dietary patterns, physical training, and chronoactivity

Although only a few studies have investigated the impact of dietary patterns on HF outcomes so far, these strategies may affect circadian rhythms and cardiac metabolic efficiency [111]. Intermittent fasting provided beneficial consequences in different clinical settings, including HF [112]. Though the precise mechanisms are still to be clarified, the benefits of intermittent fasting may be related to the increase in ketogenesis [113], the resetting of the microbiome [86], and the realignment of the circadian metabolic rhythms within cardiac cells [112].

Physical training is a fundamental therapeutic option in the HF armamentarium [114]. In a landmark study, Coats et al. described how a controlled exercise program improved cardiorespiratory functions, physical tolerance, and circadian autonomic balance in HF patients [115]. Accordingly, 6-month exercise-based cardiac rehabilitation reduced sympathetic activity both at rest and during exercises, by the desensitization of the sympathoexcitatory metaboreflex [116], hyperactive in HF patients [87]. Exercise training may also reduce the detrimental effects of other circadian disruptors, such as altering body temperature or sleep quality/duration [117]. Though long-lasting physical exercise may affect clock gene expression in the skeletal muscles, the clinical implications of this resetting are unknown [118].

The timing of physical activity (i.e., “chronoactivity”) seems to affect the cardiovascular system, too. In a study including over 80,000 individuals, morning physical activity lowered the risk of incident cardiovascular diseases [119]. On the other hand, evening exercise was associated with worse sleep quality, due to altered body temperature, in some studies, but not in others [120]. Since exercise acts as one of the strongest *zeitgebers* [21], future research should investigate whether regular training and which time of the day is more beneficial to improve circadian homeostasis in HF patients.

### Peripheral clock modulation

Considering the role of peripheral clocks, targeting the related pathways may be a potential therapeutic strategy. In this regard, lifestyle interventions aimed at resynchronizing the timing of endogenous and exogenous *zeitgebers* (e.g., light/dark, sleep/wake, feed/fast, and activity/rest) are expected to optimize metabolic processes and body efficiency, by modulating protein synthesis, activity, and degradation over time. Nevertheless, the impact of this kind of intervention in HF is unknown since no dedicated studies have been conducted so far.

Of note, the direct modulation of the peripheral clock has been proposed to anticipate pathological remodeling and HF development. In a pressure-overload mouse model, the administration of REV-ERBα agonist (SR9009), prevented cardiac remodeling and reduced disease progression in the case of established chronic HF [121]. Similar findings were reported in mouse models of ischemia–reperfusion myocardial injury, in which the short-term administration of SR9009 was associated with improved left ventricular function and survival rate [122, 123]. Considering the pleiotropic roles of this molecule, the precise mechanism of action underlying its beneficial effects is unclear, though the transcriptional modulation of fibrotic and inflammatory pathways was identified as possible mediators [122, 123]. Future research is then expected to verify the safety and the potential applicability of these encouraging findings in the clinical setting.

## Current limitations and future directions

Despite the growing recognition of the importance of chronobiology in HF pathophysiology, its integration into clinical practice remains limited. Key areas of research are still poorly understood, while practical challenges continue to hinder the clinical application of chronobiological insights. The main gaps and limitations to be addressed in the near future are as follows:

- The role of peripheral clocks in HF incidence and progression is unclear. Standardized multi-omics approaches could help to identify the involved transcriptional, translational, and post-translational mechanisms, uncovering new treatment avenues based on the modulation of peripheral clocks through key zeitgebers such as sleep/wake cycles, light/dark exposure, diet, physical activity, and body temperature.
- The chronobiological dynamics of neurohormonal systems are only partially understood. Further investigation should focus on how abnormal circadian rhythms influence autonomic imbalance and vice versa, testing the potentialities of time-adjusted drug administration and neuromodulation approaches to prevent and treat HF.
- Currently, knowledge about the role of chronobiology in HF pathophysiology mainly derives for preclinical research. Well-designed observational and interventional clinical studies, using standardized approaches, are needed to confirm these findings in the clinical scenario.
- Chronobiological mechanisms are complex, and their importance is often neglected in the clinical settings. Integrating chronobiology into healthcare education could equip clinicians with the knowledge needed to understand how circadian rhythms affect HF incidence, progression, outcomes, and drug efficacy, thus facilitating clinical application.
- Implementing chronobiology in HF care may require collaboration among cardiologists, physiologists, molecular biologists, and sleep medicine experts. Since such collaboration is often unfeasible in routine practice, establishing multidisciplinary HF teams could help bridge this gap and promote the use of chronobiological insights in daily clinical care.

## Conclusions

Despite advancements in therapy, the prevalence of HF is still rising, and its associated morbidity and mortality are only partially counterbalanced by current therapies. While the impact of sleep disorders and neurohormonal activation on HF patients has been extensively studied, the importance of chronobiology has only recently gained recognition. Mounting evidence suggests that abnormal rhythms in neurohormonal and cardiovascular control due to sleep/breathing disorders and sedentary lifestyles may contribute to HF pathophysiology. To optimize therapeutic interventions, it is however essential to gain a deeper understanding of the mechanisms underlying the disrupted chronobiology of the cardiovascular and neurohormonal systems.

In parallel with ongoing research, to prompt an early integration of chronobiological principles into clinical practice, it is critical for clinicians to recognize the importance of circadian rhythms in HF prevention and management. Practical steps such as screening for circadian misalignment and encouraging patients to maintain regular sleep–wake cycles, adopt healthy diets, and engage in consistent physical activity can help realign disrupted circadian rhythms and improve HF outcomes. These time-tailored lifestyle interventions represent an initial and accessible approach that can be implemented in routine clinical care, while awaiting more advanced therapeutic protocols derived from future well-designed chronobiology research in HF patients.

## Data Availability

No datasets were generated or analysed during the current study.

## Abbreviations

BMAL1: Brain, and muscle Arnt-like protein 1
CA: Central apneas
CLOCK: Circadian locomotor output cycle kaput
CRY: Cryptochrome
HF: Heart failure
OSA: Obstructive sleep apneas
PER: Period
Rev-erbα: Orphan nuclear-receptor gene
RORα/β: Retinoic acid receptor-related orphan receptors α/β
SCN: Suprachiasmatic nucleus
